# Prevalence of pemphigus and pemphigoid autoantibodies in the general population

**DOI:** 10.1186/s13023-015-0278-x

**Published:** 2015-05-15

**Authors:** Wiebke Prüßmann, Jasper Prüßmann, Hiroshi Koga, Andreas Recke, Hiroaki Iwata, David Juhl, Siegfried Görg, Reinhard Henschler, Takashi Hashimoto, Enno Schmidt, Detlef Zillikens, Saleh M. Ibrahim, Ralf J. Ludwig

**Affiliations:** Department of Dermatology, University of Lübeck, Lübeck, Germany; Lübeck Institute of Experimental Dermatology (LIED), University of Lübeck, Lübeck, Germany; Institute of Transfusion Medicine, University Hospital of Schleswig-Holstein, Lübeck and Kiel, Germany; Transfusion Medicine, Cellular Therapeutics and Hemostaseology, Clinics of the Ludwigs-Maximilians-University Munich, Munich, Germany; Department of Dermatology, Kurume University School of Medicine, and Kurume University Institute of Cutaneous Cell Biology, Kurume, Japan; Department of Dermatology, Hokkaido University Graduate School of Medicine, Sapporo, Japan

**Keywords:** Autoimmunity, Skin, Type XVII collagen, BP180, Desmoglein, Pemphigoid, Pemphigus

## Abstract

**Background:**

Mucocutaneous blistering is characteristic of autoimmune bullous dermatoses (AIBD). Blisters are caused by autoantibodies directed against structural components of the skin. Hence, detection of specific autoantibodies has become a hallmark for AIBD diagnosis. Studies on prevalence of AIBD autoantibodies in healthy individuals yielded contradictory results.

**Methods:**

To clarify this, samples from 7063 blood donors were tested for presence of anti-BP180-NC16A, anti-BP230 and anti-Dsg1/3 IgG by indirect immunofluorescence (IF) microscopy using a biochip.

**Results:**

Cumulative prevalence of these autoantibodies was 0.9 % (CI: 0.7–1.1 %), with anti-BP180-NC16A IgG being most prevalent. Validation of IF findings using ELISA confirmed presence of autoantibodies in 7/15 (anti-Dsg1), 6/7 (anti-Dsg3), 35/37 (anti-BP180-NC16A) and 2/3 (anti-BP230) cases. Moreover, in 16 samples, anti-BP180-NC16A autoantibody concentrations exceeded the cut-off for the diagnosis of bullous pemphigoid. Interestingly, these anti-BP180-NC16A autoantibodies from healthy individuals formed immune complexes with recombinant antigen and dose-dependently activated neutrophils *in vitro*. However, fine-epitope mapping within NC16A showed a different binding pattern of anti-BP180-NC16A autoantibodies from healthy individuals compared to bullous pemphigoid patients, while IgG subclasses were identical.

**Conclusions:**

Collectively, we here report a low prevalence of AIBD autoantibodies in a large cohort of healthy individuals. Furthermore, functional analysis shows differences between autoantibodies from healthy donors and AIBD patients.

**Electronic supplementary material:**

The online version of this article (doi:10.1186/s13023-015-0278-x) contains supplementary material, which is available to authorized users.

## Background

Autoimmune bullous dermatoses (AIBD) are clinically characterized by chronic mucocutaneous blistering, leading to severe morbidity and increased mortality [1–4]. Blister formation is directly or indirectly caused by autoantibodies binding to structural proteins of the skin [5, 6]. Depending on the location of the blister and the targeted autoantigens, AIBD can be classified as pemphigus and pemphigoid disease, epidermolysis bullosa acquisita (EBA) and dermatitis herpetiformis [7, 8]. Epidemiological studies have documented the incidence of AIBD in several geographic regions. In central Europe, bullous pemphigoid (BP) had the highest incidence, with 6.1 to 42.8 cases per million persons per year [1, 3, 4, 9–13]. For pemphigus disease, including pemphigus vulgaris (PV) and pemphigus foliaceus (PF), the reported incidence ranged from 0.6 to 6.8 cases per million persons per year [1, 14–16].

For other autoimmune diseases, studies analyzed serum samples obtained from individuals before they received a diagnosis of systemic lupus erythematousus (SLE) or rheumatoid arthritis (RA). These studies clearly demonstrated the presence of autoantibodies several years before diagnosis [17, 18]. Derived from these findings, one may assume that autoantibodies in AIBD also predate the onset of the corresponding disease. However, based on the combined yearly incidence of all AIBD of 0.005 %, to conduct such an investigation with 50–100 AIBD patients would require a predated serum collection of 1–2 million people. In addition, clinically healthy individuals have not been systematically investigated for the presence of autoantibodies to structural proteins of the skin and the reported autoantibody prevalence is contradictory. For example, the following autoantibody prevalence rates in healthy populations have been reported: 0–0.7 % for autoantibodies to desmoglein 1 (Dsg) (PF autoantigen); 0–0.2 % for anti-Dsg3 (PV autoantigen); 0-2 % for anti-BP180-NC16A (BP autoantigen); and 0-7 % for anti-BP230 (BP autoantigen) antibodies (Table 1). Therefore, in this study, we aimed at determining the prevalence of autoantibodies against desmosomal and hemidesmosomal structural proteins in a large population of healthy blood donors. In addition, the potential pathogenic relevance of the detected autoantibodies was evaluated.

**Table 1:** Previously reported prevalence rates of autoantibodies to structural proteins of the skin

Antigen	Population (n)	Prevalence	Reference
Dsg1	Normal subjects (53)	0.0 %	[35]
	Blood donors (401)	0.7 %	[20]
Dsg3	Normal subjects (53)	0.0 %	[35]
	Blood donors (401)	0.2 %	[20]
BP180*	Healthy volunteers (47)	0.0 %	[36]
	Blood donors (494)	2.0 %	[19]
	Normal subjects (336)	1.5 %	[37]
	Healthy subjects (61)	0.0 %	[38]
BP230	Normal controls (109)	0.0 %	[39]
	Healthy controls (56)	7.0 %	[40]
	Blood donors (483)	2.1 %	[41]

*to BP180-NC16A if not otherwise noted

## Methods

### Blood Donors

This study included 7063 normal blood donors from the Institutes for Transfusion Medicine Lübeck, Kiel and Frankfurt between August 2010 and March 2011. All samples were anonymized immediately after blood drawing to comply with requirements by the ethics committees. To avoid duplicate testing of the same person, blood samples from all frequent donors were collected within eight weeks, which is the shortest possible donation interval for men. Further collection was restricted to first-time donors. All plasma aliquots were stored at −20 °C until further testing. All participants signed an informed consent. The study was performed according to the principles of the Declaration of Helsinki and was approved by the local ethics committees (10–094, the ethics committee of the University of Lübeck).

### Autoantibody screening

Plasma samples from all 7063 donors were analyzed for the presence of pemphigus- and pemphigoid-related antibodies with a commercial indirect immunofluorescence (IF) assay (dermatology-mosaic 7, EUROIMMUN AG, Lübeck, Germany) at a 1:10 dilution. The assay included the following substrates: primate esophagus, primate salt split skin, recombinant tetrameric BP180-NC16A and transfected HEK293 cells that express recombinant BP230, Dsg1 or Dsg3. Specific fluorescence (Additional file 1) at a dilution of 1:10 was considered positive, as advised in the instruction manual. Indirect IF microscopy-positive samples were subsequently evaluated for specific antibodies (IgG) with enzyme-linked immunosorbent assays (ELISA, Anti-BP180-NC16A-ELISA, Anti-BP230-CF-ELISA, Anti-Dsg-1-ELISA, Anti-Dsg3-ELISA, all EUROIMMUN AG) [19, 20]. These different ELISAs share a detection threshold of 0.5 to 0.6 RU/ml and linearity from at least 10 to 199 RU/ml. Calibrators with 2, 20 and 200 RU/ml were used to quantify the measured extinction rates with computer software. For values below 1 RU/ml, the software is technically unable to calculate exact values, so that values for these samples are set “<1 RU/ml” and were considered negative. To consolidate the read out from indirect IF microscopy, a comparable number of indirect IF microscopy-negative samples were also tested for each of the relevant antigens by ELISA.

### ROS release from human PMN

ROS release from immune complex-activated human PMN was performed as described in [21] with some modifications. Briefly, 500 ng of tetrameric form of the NC16A domain of BP180 recombinant protein [19] was coated each wells followed by addition of 1:10 diluted plasma samples and incubation for 1 h. After washing well, PMN was added to each well. Each sample was tested with PMNs isolated from 3 healthy donors in duplicate.

### Determination of IgG subclasses

For determination of BP180-NC16A IgG1-4 reactivity, a commercial BP180-NC16A ELISA was used [19] and adapted to detect IgG1-4 antibodies. After incubation with human plasma, ELISA plates were incubated with biotin-conjugated mouse anti-human IgG1, IgG2, IgG3 and IgG4 antibodies (Invitrogen, Camarillo, CA), followed by incubation with peroxidase-conjugated donkey anti-mouse IgG antibody (minimal cross-reaction to serum proteins of other species, Jackson ImmunoResearch Laboratories, West Grove, PA). As we found low BP180-NC16A-IgG4 reactivity, the sensitivity of the protocol was evaluated using sera from pemphigus patients. Here, we frequently detected anti-Dsg3 IgG4 autoantibodies (not shown). The cut-off values were determined as mean value +3SD from 10 healthy blood donors.

### Fine epitope mapping of anti-BP180-NC16A reactivity

For determination of the reactivity of BP180-NC16A+ sera to BP180-NC16A2-3 (Biotin-RSILPYGDSMDRIEKDRLQGMAPAAGADL, Peptides and elephants GmbH, Potsdam, Germany), we followed previously described protocols [22]. Briefly, streptavidin-coupled microtiter plates were coated for 1 h on a shaker with 200 ng of the biotinylated synthetic peptide. After washing the plate, sera diluted 1:200 in PBST were added to the plate and incubated for 1 h, followed by HRP-conjugated anti-human IgG incubation for 1 h. Bound IgG were visualized by 1-Step Turbo TMB-ELISA (Pierce, Rockford, IL) and measured at 450 nm. The cut-off value was determined as mean value +3SD from 10 healthy blood donors. Each sample was tested in duplicate.

### Dsg3 internalization assay

HaCaT cells were cultured with keratinocyte growth medium 2 (PromoCell GmbH, Heidelberg, Germany). At confluence, the calcium concentration of the medium was adjusted to 1.5 mM and incubated for 24 h. Then, 1 mg/ml of IgG purified from healthy donors (Intratect®, Biotest Pharma GmbH, Dreieich, Germany), PV patients or healthy donors with or without anti-Dsg3 antibodies at dilution indicated at the Figure legends was added to HaCaT cells and incubated at 37 °C in a CO2 incubator for 24 h. After incubation, the cells were fixed and permeabilized followed by blocking with 1 % BSA and 10 % normal goat serum. Dsg3 was detected by mouse anti-Dsg3 antibody (Acris antibodies, Herford, Germany) and Cy3-goat anti-mouse IgG (Jackson ImmunoResearch, West Grove, PA). Fluorescence images were obtained using a fluorescence microscope (BZ-9000, Keyence, Osaka, Japan). To determine the anti-Dsg3 antibody index value, all samples were adjusted 15 mg/ml and measured by anti-Dsg3-ELISA (EUROIMMUN AG).

### Statistical analysis

Statistical analysis was performed with Gnu R (version 2.14.1, http://www.r-project.org) together with package “epitools” (version 0.5–6) for calculation of Wilson confidence intervals for binomial counts. ELISA values of IIF-positive and IIF-negative samples were compared by the Wilcoxon rank-sum test with continuity correction using SigmaPlot (Version 12).

## Results

### Low prevalence of pemphigus and pemphigoid autoantibodies in the general population

For this study, plasma samples from 7063 healthy blood donors were collected and tested for reactivity with autoantigens in AIBD using indirect IF microscopy with antigens coated on a biochip. Autoantibody reactivity in positively rated samples was validated using commercially available ELISA systems. From these 7063 samples, a total of 62 samples reacted with at least one of the recombinant substrates used for indirect IF microscopy, amounting to a 0.88 % (95 % CI: 0.66–1.10 %) overall prevalence of autoantibodies to desmosomal and/or hemidesmosomal structural proteins of the skin. Of these, 0.52 % (95 % CI: 0.36-0.69 %) reacted with BP180-NC16A, 0.04 % (95 % CI: 0.00–0.09 %) with BP230, 0.21 % (95 % CI: 0.11–0.32 %) with Dsg1 and 0.10 % (95 % CI: 0.03–0.17 %) with Dsg3 (Table 2). Although recombinant antigen reactivity was observed in indirect IF microscopy, visible esophagus tissue staining was frequently absent. In detail, only 5 % of all BP180/230-positive samples showed a basement membrane pattern, whereas 55 % of all Dsg1/3-positive samples showed a desmosomal pattern irrespective of the measured ELISA values. Overall, 0.31 % (CI: 0.18–0.44 %) of healthy blood donors had autoantibodies against pemphigus antigens, and 0.52 % (95 % CI: 0.36–0.69 %) showed reactivity with BP180-NC16A, the relevant pathogenic epitope of BP [23–25].

**Table 2:** Prevalence of pemphigus and pemphigoid autoantibodies in healthy blood donors

Antigen	IIFM positive* [n]	IIFM positive* [%]	ELISA**		
			Negative [n]	Below diagnostic cut-off [n]	Above diagnostic cut-off (≥20 RU/ml) [n]
Dsg1^#^	15	0.21 (0.11-0.32)	8	6	1
Dsg3^#^	7	0.10 (0.03-0.17)	1	6	0
BP180-NC16A^§^	37	0.52 (0.36-0.69)	2	19	16
BP230^#^	3	0.04 (0.00-0.09)	1	2	0

*From a total of 7063 samples. Numbers in parentheses correspond to a 95 % confidence interval. This table contains double positives. **All IIF-positive samples were included (15 for Dsg1, 7 for Dsg3, 37 for BP180-NC16A, 3 for BP230). ^#^Transfected HEK cells expressing recombinant antigen. ^§^Purified spotted protein. Abbreviations used: IIFM, indirect immunofluorescence microscopy

### Validation of detected autoantibody prevalence

**Fig. 1:**
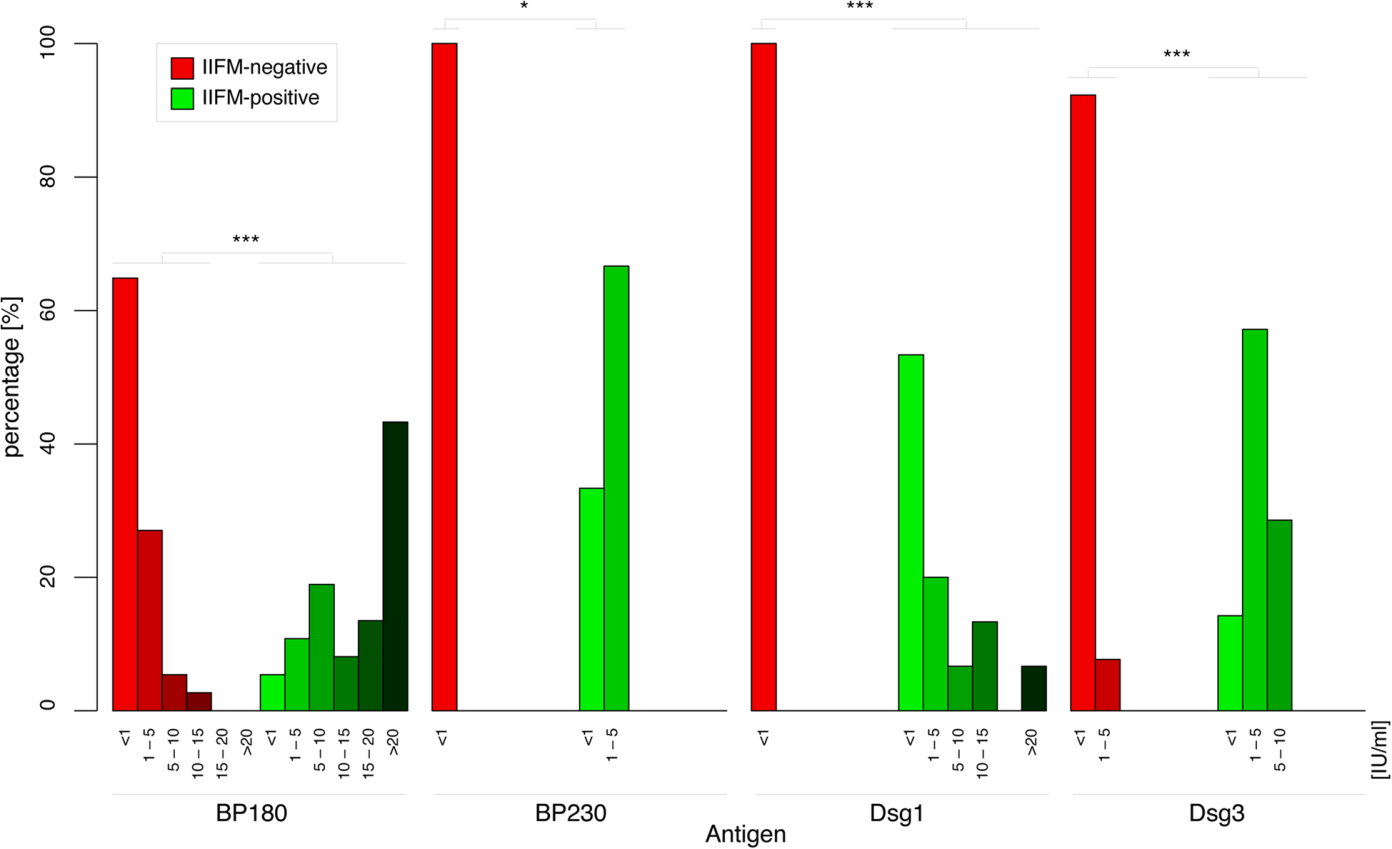
ELISA values of indirect IF microscopy-positive and indirect IF microscopy-negative samples. Percentage of indirect IF microscopy (IIFM)-positive (green) and indirect IF-microscopy negative (red) samples according to the measured ELISA values. Anti-BP180-NC16A-, anti-BP230-, anti-Dsg1-and anti-Dsg3-positive samples have significantly higher ELISA values than antibody-negative samples by the Wilcoxon rank-sum test with continuity correction. All indirect IF microscopy-positive samples [n: indirect IF microscopy-negative samples] were included: 15 [27] for Dsg1, 7 [13] for Dsg3, 37 [37] for BP180-NC16A, 3 [9] for BP230. *p < 0.05, ***p < 0.001 (comparing indirect IF microscopy-negative with indirect IF microscopy-positive samples). Abbreviations: IIFM: indirect IF microscopy

Next, positively rated samples (BP180-NC16A, BP230 and Dsg 1/3 reactivity) were validated by ELISA (Fig. 1, Table 2). Overall, anti-BP180-NC16A-, anti-BP230-, anti-Dsg1- and anti-Dsg3 IF-positive samples had significantly higher ELISA values than IF-negative samples. Remarkably, BP180-NC16A ELISA reactivity was detected in 35 of 37 indirect IF microscopy-positive samples, and 16 of those were above the cut-off value for the diagnosis of BP (20 RU/ml). Regarding BP230 indirect IF microscopy-positive samples, 2 of 3 had anti-BP230 antibodies detectable by ELISA. In 7 of 15 Dsg1 indirect IF microscopy-positive samples reactivity was also detected by ELISA, while 8 samples were negative. Of 7 Dsg3 indirect IF microscopy-positive samples, 6 had autoantibodies that were detectable by ELISA.

### BP180-NC16A autoantibodies from healthy blood donors form immune complexes and dose-dependently activate neutrophils

**Fig. 2:**
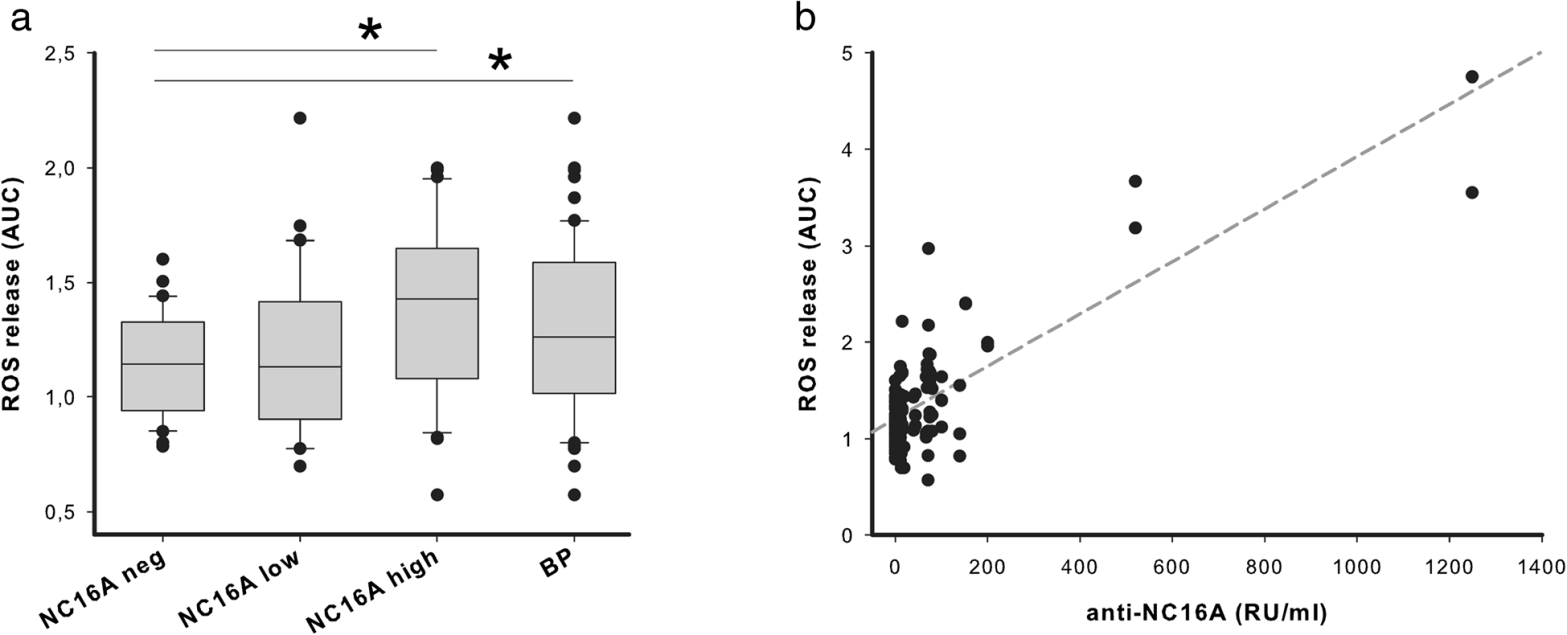
Anti-BP180-NC16A autoantibodies from healthy individuals induce ROS release from neutrophils. (**a**) ROS release of immune complex-activated PMN in relation to cells incubated with antigen only. Owing to the non-parametric distribution, the data are presented as the median (line), 25/75-percentile (boxes), 5/95-percentiles (error bars) and outliers (dots). *p < 0.05 (ANOVA on ranks followed by Dunn’s method for multiple comparisons versus control (NC16A-negative samples). (**b**) Concentrations of anti-BP180-NC16A IgG correlate with the amount of ROS release from PMN (r = 0.786, p < 0.001, Pearson product-moment correlation)

The binding of neutrophils to immune complexes (ICs) leads to their activation, which can be analyzed by their release of reactive oxygen species (ROS) [26]. Incubation of polymorphonuclear leukocytes (PMN) from healthy blood donors with IC of recombinant BP180-NC16A and BP patient plasma leads to a significant ROS release [21]. When recombinant BP180-NC16A was incubated with plasma from healthy blood donors, ROS release was identical to baseline (antigen only) when BP180-NC16A-negative or -positive samples (below the diagnostic cut-off) were added. If, however BP180-NC16A-positive (above the diagnostic cut-off) plasma samples from healthy blood donors were used, this led to an increase in ROS release similar to the effect of samples from BP patients (Fig. 2a). Overall, the concentration of anti-BP180-NC16A IgG correlated with the ROS release from PMN (Fig. 2b), indicating that anti-BP180-NC16A IgG from healthy blood donors is able to activate PMN *in vitro*.

### BP180-NC16A autoantibodies detected in healthy blood donors are predominantly of the IgG3 subclass

In endemic pemphigus, a shift from IgG1 to IgG4 autoantibodies has been linked to pathogenesis [27]. To test whether the IgG subclasses differ among anti-BP180-NC16A IgG from healthy individuals, as opposed to BP patients, a modified BP180-NC16A ELISA was performed. As shown in Table 3, the frequencies of all IgG subclasses for anti-BP180-NC16A antibodies were lower in the NC16A-IgG positive blood donors compared to BP patients. This was most pronounced for IgG2. Anti-BP180-NC16A IgG3 was found most often in the BP180-NC16A IgG-positive healthy blood donors (Table 3).

**Table 3:** Subclasses of anti-BP180-NC16A IgG in healthy blood donors and bullous pemphigoid patients

	IgG1	IgG2	IgG3	IgG4
Healthy, < 20 RU/ml*	0/10	1/10	5/10	0/10
Healthy, ≥ 20 RU/ml*	2/10	1/10	4/10	1/10
BP patients	4/10	7/10	7/10	0/10
Healthy blood donors	0/10	0/10	0/10	0/10

*value of BP180-NC16A ELISA

### Epitopes targeted by BP180-NC16A autoantibodies detected in healthy blood donors do not overlap with those predominantly recognized by BP patients

**Fig. 3:**
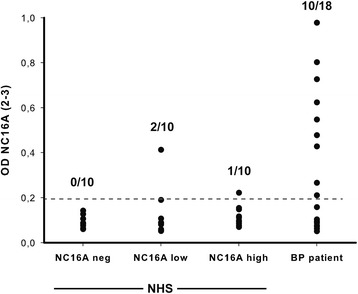
Anti-BP180-NC16A autoantibodies from healthy individuals bind to different epitopes within the NC16A domain. Samples from the indicated groups were tested for their IgG reactivity against NC16A2-3 (RSILPYGDSMDRIEKDRLQGMAPAAGADL). As reported previously [29, 30], high reactivity with this epitope was observed in BP patients. By contrast, only a minority of samples from healthy individuals with IgG reactivity against NC16A showed reactivity with this epitope. Abbreviations: NHS: normal human sera

As intramolecular epitope spreading towards certain epitopes of Dsg1 has been reported to lead to clinical disease manifestation in endemic pemphigus [28], we next compared the fine epitope specificity of BP180-NC16A IgG from healthy individuals and BP patients. The anti-BP180-NC16A IgG reactivity in BP patients had been shown to cluster at epitopes located within the BP180-NC16A2-3 region [29, 30]. In our cohort of BP patients, 10/18 (56 %) samples reacted with this epitope, confirming these previous findings. Interestingly, BP180-NC16A-positive samples from healthy blood donors rarely bound to this epitope. In detail, only 2/10 samples below the diagnostic cut-off, and 1/10 samples above the diagnostic cut-off, recognized this epitope (Fig. 3).

### IgG from healthy individuals with antibody reactivity to Dsg3 do not induce Dsg3 internalization *in vitro*

**Fig. 4:**
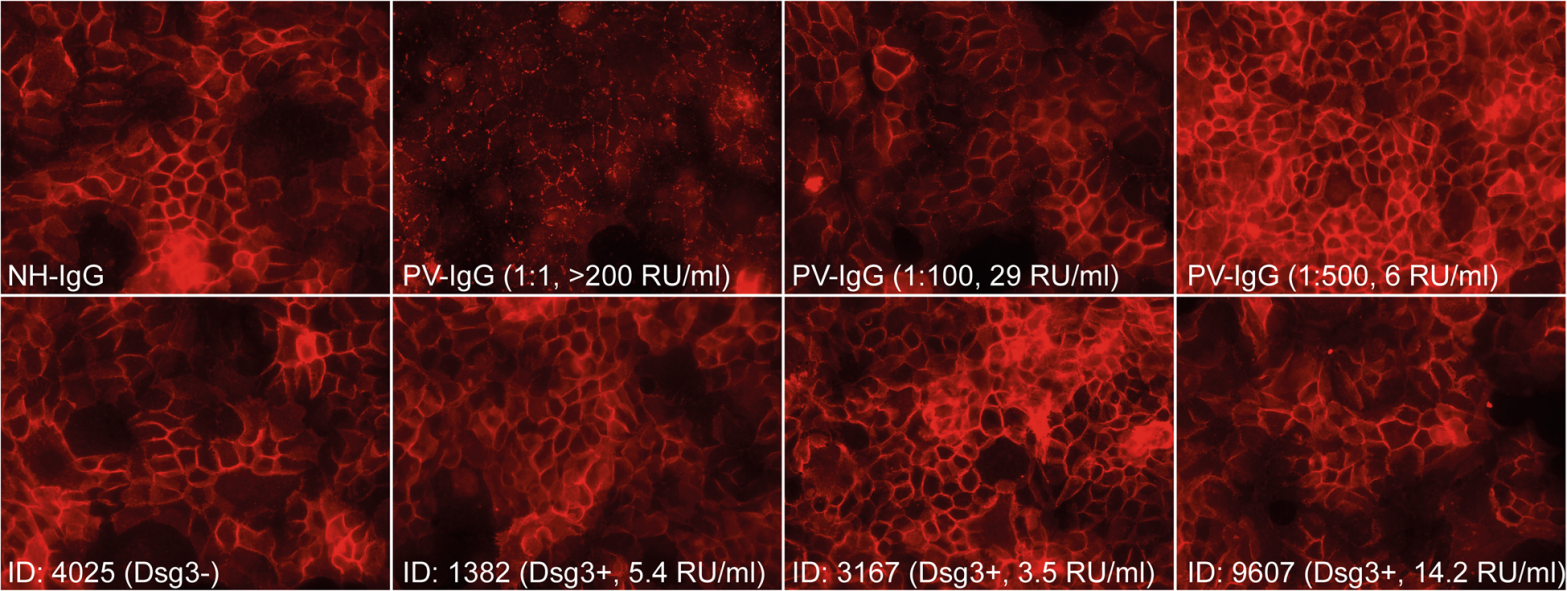
Anti-Dsg3 autoantibodies detected in healthy blood donors do not lead to Dsg3 internalization *in vitro*. HaCaT cells were incubated with IgG from healthy individuals (NH-IgG), IgG from a pemphigus vulgaris patient (PV-IgG) or with Dsg3-positive plasma samples from healthy blood donors (samples #1382, #3167 and #9607), as well as with a Dsg3-negative sample (sample #4025). For all experimental conditions (*n* = 3), the IgG concentration was 1 mg/ml (with the exception of the diluted PV-IgG samples). While NH-IgG and 4025 did not induce Dsg3 internalization, PV-IgG at 1:1 and 1:100 dilutions led to Dsg3 internalization. At low dilutions, PV-IgG and all tested Dsg3-positive samples from healthy blood donors did not show any effects on Dsg3 internalization

We also tested for potential pathogenicity of the detected anti-Dsg3 IgG reactivity in the healthy blood donors. However, none of the tested samples induced the internalization of Dsg3 when incubated with HaCaT cells, as opposed to IgG from a pemphigus vulgaris patient (Fig. 4). This lack of pathogenicity is most likely due to the low anti-Dsg3 IgG concentrations in these samples.

### Discussion

Our data document an overall low prevalence of pemphigus and bullous pemphigoid IgG autoantibodies in a collection of 7063 blood donors. To date, this is the largest population studied for pemphigus and pemphigoid autoantibodies. In line with BP being the most common AIBD in central European countries, we found BP-associated autoantibodies more frequently than pemphigus-associated autoantibodies. Compared to the reported prevalence of pemphigus (0.004 %) and pemphigoid (0.016 %) in Denmark [31], the autoantibody prevalence reported here is 25 or 37-fold higher. Hence, one may assume that in BP and pemphigus, as reported for other autoimmune diseases [17, 18], autoantibodies predate the onset of clinical disease. Based on the prevalence of pemphigus and pemphigoid antibodies in healthy individuals and the prevalence of AIBD, every 25^th^ individual with autoantibodies against pemphigus antigens is likely to develop pemphigus; similarly, every 37^th^ healthy individual with anti-BP180-NC16A IgG reactivity would be expected to develop BP. This process would be initiated by the break of tolerance and the generation of autoantibodies to structural proteins of the skin. Subsequently, an AIBD will become clinically manifest in only a minority of affected autoantibody-positive healthy individuals. In the future, additional biomarkers will hopefully allow clinicians to distinguish between “resistant” antibody-positive individuals and those who are prone to developing overt clinical disease.

In contrast to the detection of other autoantibodies in healthy individuals, e.g., ANA [17], the potential pathogenic activity of anti-BP180-NC16A IgG and Dsg1/3 can be tested [6]. As shown here, anti-BP180-NC16A IgG from healthy blood donors induces neutrophil activation *in vitro*. Because neutrophil activation is a key requirement for BP pathogenesis [32], the blister-inducing ability of these autoantibodies can be assumed. Furthermore, anti-BP180-NC16A IgG subclasses were identical between healthy blood donors and BP patients (Table 3). Our result that no IgG4 anti-BP180-NC16A antibodies were detected in BP patients seemingly contradicts a previous report [33], although it might be due to the small number of BP serum samples included in our current study. In addition, the fine mapping of the autoantibody response in our samples revealed differences in the cohort of healthy blood donors compared to BP patients (Fig. 3). This is similar to observations made for endemic pemphigus with autoantibodies directed against Dsg1. Epidemiologic studies demonstrated a shift from IgG1 to IgG4 autoantibodies [34] and intramolecular epitope spreading towards certain epitopes [28] upon development of skin blisters in endemic pemphigus foliaceus. The anti-Dsg3-positive sera from our collection of healthy blood donors demonstrated a lack of pathogenic potential of these autoantibodies, which is most likely due to the low concentration of autoantibodies in the samples (Fig. 4).

Our results on the prevalence of autoantibodies to structural proteins of the skin also resolve the discrepancies among the reports on this topic to this point. Regarding Dsg1 antibodies, the reported prevalences in healthy individuals ranged from 0 % to 0.7 %. A study by Ishii et al. included 53 healthy control sera, and none reacted to baculovirus-expressed Dsg1 in an ELISA [35]. In a study by Schmidt et al., 401 healthy blood donors were included, and circulating Dsg1 antibodies were detected using ELISA with the Dsg1 ectodomain, expressed in a human cell line, as a substrate. There, 3 of the 401 healthy controls had ELISA values above the diagnostic cut-off for pemphigus [20]. We showed here that 0.1 % of healthy individuals have detectable Dsg1 autoantibodies, which were detected by indirect IF microscopy using Dsg1-expressing cells as a substrate. Validation of the 15 indirect IF-positive samples by ELISA showed reactivity in 7 of the samples. However, with the exception of one sample, the values were below the diagnostic cut-off value. Compared with the study of Schmidt et al. we found far fewer Dsg1-reactive samples. This may be explained by the fact that Schmidt et al. defined Dsg1 positivity sorely by ELISA. In contrast, in our study, only those sera were considered positive that reacted with Dsg1 by both indirect IF microscopy and ELISA. Similar considerations also hold true for the other autoantigens in AIBD tested within our study (Table 1).

## Conclusion

In summary, we here document the prevalence of pemphigus and pemphigoid autoantibodies in a large cohort of healthy blood donors. The comparison of the autoantibody prevalence and the prevalence of the corresponding AIBD may suggest that these diseases develop over a prolonged period of time.

## Additional file

Additional file 1:
**Examples of indirect immunofluorescence (IF) patterns observed in the study.** Screening for antibodies associated with autoimmune skin blistering diseases was performed using biochip-based indirect IF assay. All images show picture details of positive samples from our cohort.
