# Supplementary figures and images for: Prevalence of pemphigus and pemphigoid autoantibodies in the general population
Source: Orphanet J Rare Dis. 2015 May 15;10:63. doi: 10.1186/s13023-015-0278-x (PMC4436865; doi:10.1186/s13023-015-0278-x)

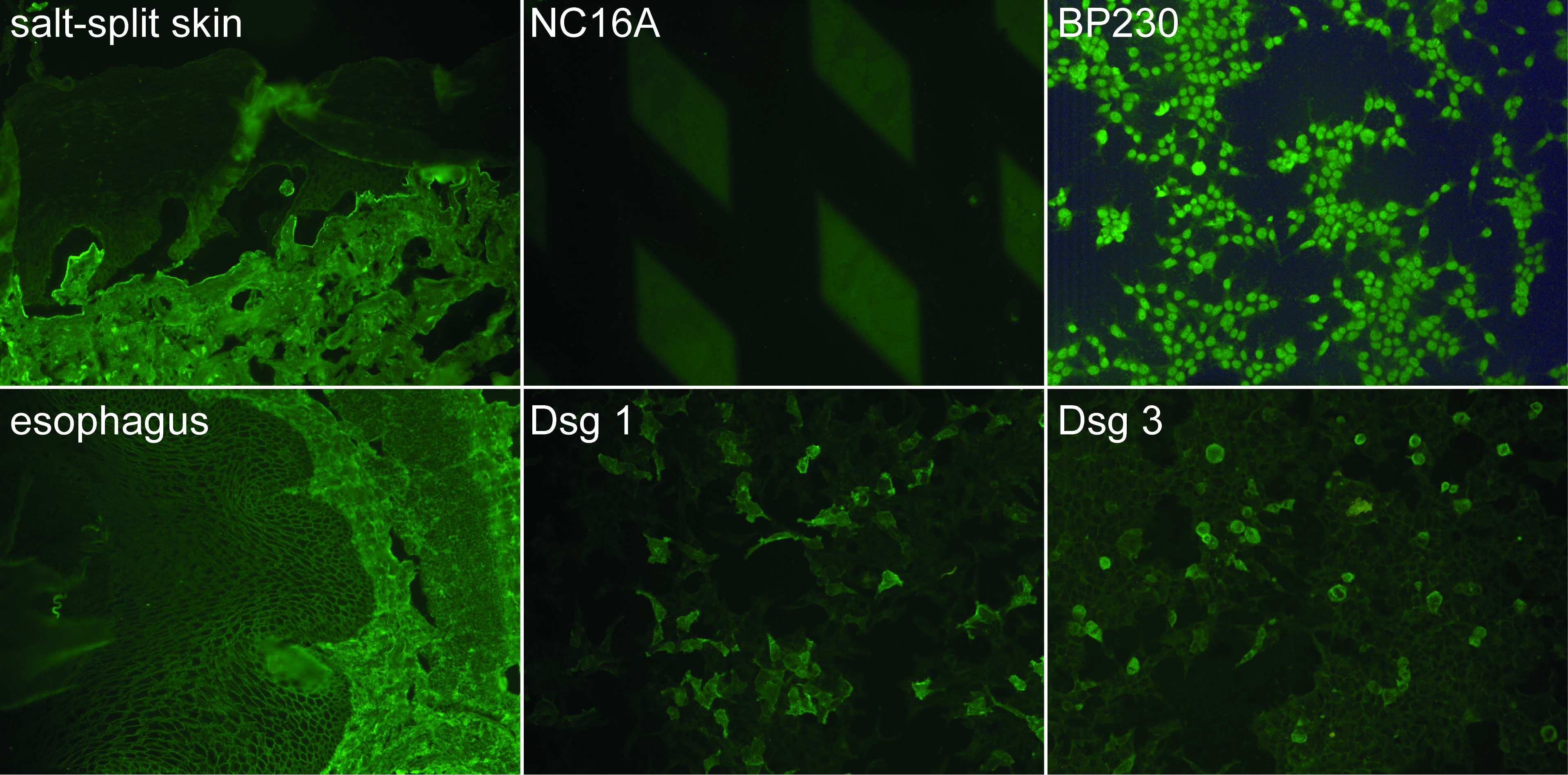

Supplement: Additional file 1: — Examples of indirect immunofluorescence (IF) patterns observed in the study. Screening for antibodies associated with autoimmune skin blistering diseases was performed using biochip-based indirect IF assay. All images show picture details of positive samples from our cohort. [file 13023_2015_278_MOESM1_ESM.tif]
